# Supplementary material for: Exploring the feasibility of a network of organizations for pain rehabilitation: What are the lessons learned?
Source: PLoS One. 2022 Sep 15;17(9):e0273030. doi: 10.1371/journal.pone.0273030 (PMC9477302; doi:10.1371/journal.pone.0273030)
Supplement: S1 Checklist — (PDF) [file pone.0273030.s001.pdf]

Consolidated criteria for reporting qualitative studies (COREQ): 32-item checklist

No	Item	Guide questions/description
**Domain 1: Research team and reflexivity**		
Personal Characteristics		
1. **P6 (L.174-176)**	Interviewer/facilitator	Which author/s conducted the interview or focus group?
2. **P6 (L.175)**	Credentials	What were the researcher's credentials? E.g. PhD, MD
3. . **P6 (L.175-176)**	Occupation	What was their occupation at the time of the study?
4. **CL- female GB-male**	Gender	Was the researcher male or female?
5. **P6 (L.175-176)**	Experience and training	What experience or training did the researcher have?
Relationship with participants		
6. **P6. (L173-174)**	Relationship established	Was a relationship established prior to study commencement?

No	Item	Guide questions/description
**7. CL had contact with HCPs during meetings and knew her. GB was independent, this was introduced at the start of the focus groups. Patients did not know the interviewers**	Participant knowledge of the interviewer	What did the participants know about the researcher? e.g. personal goals, reasons for doing the research
**8. P5 (L.121) P6 (L.173-174)**	Interviewer characteristics	What characteristics were reported about the interviewer/facilitator? e.g. Bias, assumptions, reasons and interests in the research topic
**Domain 2: study design**		
Theoretical framework		
**9. P8 (L.216)**	Methodological orientation and Theory	What methodological orientation was stated to underpin the study? e.g. grounded theory, discourse analysis, ethnography, phenomenology, content analysis

No	Item	Guide questions/description
Participant selection		
10. **P5-6 (L.138-151)**<br>**P6 (L.159)**	Sampling	How were participants selected? e.g. purposive, convenience, consecutive, snowball
11. **P6 (L.159-160)**	Method of approach	How were participants approached? e.g. face-to-face, telephone, mail, email
12. **P5 (L.142-143)**<br>**P6 (L.148-151)**<br>**P6 (L.159-160)**	Sample size	How many participants were in the study?
13. **P5 (L.143-145)**<br>**P6 (L.151)**<br>**P6 (L.159-160)**	Non-participation	How many people refused to participate or dropped out? Reasons?
Setting		
14. **P6. (L.177-179)**	Setting of data collection	Where was the data collected? e.g. home, clinic, workplace
15. **P6. (174)**	Presence of non-participants	Was anyone else present besides the participants and researchers?
16. **Table 1**	Description of sample	What are the important characteristics of the

No	Item	Guide questions/description
		sample? e.g. demographic data, date
Data collection		
17. **P6 (L.176-177)**	Interview guide	Were questions, prompts, guides provided by the authors? Was it pilot tested?
18. **No**	Repeat interviews	Were repeat interviews carried out? If yes, how many?
19. **P6 (L.176-177)**	Audio/visual recording	Did the research use audio or visual recording to collect the data?
20. **P6 (L.174-175)**	Field notes	Were field notes made during and/or after the interview or focus group?
21. **Table 1**	Duration	What was the duration of the interviews or focus group?
22. **Not applicable. Not more HCPs available**	Data saturation	Was data saturation discussed?
23. **No**	Transcripts returned	Were transcripts returned to participants for

No	Item	Guide questions/description
		comment and/or correction?
**Domain 3: analysis and findingsz**		
Data analysis		
24. **P8 (L.220-223)**	Number of data coders	How many data coders coded the data?
25. **P8 (L.218)**	Description of the coding tree	Did authors provide a description of the coding tree?
26. **P8 (L.218-222)**	Derivation of themes	Were themes identified in advance or derived from the data?
27. **P8 (L221)**	Software	What software, if applicable, was used to manage the data?
28. **P8 (L225)**	Participant checking	Did participants provide feedback on the findings?
Reporting		
29. **P10-17**	Quotations presented	Were participant quotations presented to illustrate the themes / findings? Was

No	Item	Guide questions/description
		each quotation identified? e.g. participant number
30. **Table 2**	Data and findings consistent	Was there consistency between the data presented and the findings?
31. **Table 2**	Clarity of major themes	Were major themes clearly presented in the findings?
32. **S3 Table**	Clarity of minor themes	Is there a description of diverse cases or discussion of minor themes?
