# Supplementary material for: Exploring the feasibility of a network of organizations for pain rehabilitation: What are the lessons learned?
Source: PLoS One. 2022 Sep 15;17(9):e0273030. doi: 10.1371/journal.pone.0273030 (PMC9477302; doi:10.1371/journal.pone.0273030)
Supplement: S1 Table — (PDF) [file pone.0273030.s002.pdf]

S4 Table. Overview of main barrier and facilitator nodes per CFIR domain and construct

1 Intervention Characteristic
- 1A Intervention Source
- 1B Evidence Strenght & Quality
- 1C Relative Advantage
- 1D Adaptability
- 1E Trialability
- 1F Complexity
- 1G Design Quality & Packaging
- 1H Cost

2 Outer setting
- 2A Patient Needs & Resources
- 2B Cosmopolitanism
- 2C Peer Pressure
- 2D External Policy & Incentives

3 Inner Setting
- 3A Structural Characteristics
- 3B Network & Communications
- 3C Culture
- 3D Implementation Climate
- 3D1 Tension for Change
- 3D2 Compatibility
- 3D3 Relative Priority
- 3D4 Organizational Incentives & Rewards
- 3D5 Goals and Feedback
- 3D6 Learning Climate
- 3E Readiness for Implementation
- 3E1 Leaderschip Engagement
- 3E2 Available Resources
- 3E3 Access to knowledge and information

4 Characteristics of individuals - Zorgverleners
- 4A Knowledge & Beliefs about the intervention
- 4B Self-efficacy
- 4C Individual Stage of Change
- 4D Individual Identification with Organization
- 4E Other personal Attributes

5 Process
- 5A Planning
- 5B Engaging
- 5B1 Opinion Leaders
- 5B2 Formally appointed internal implementation leaders
- 5B3 Champions
- 5B4 External Change Agents
- 5B4a Key stakeholders including staff
- 5B4b Patients or Customers
- 5C Executing
- 5D Reflecting & Evaluating

Summary 1: Within NPRL, the guidelines and treatment protocols provide consistency and transparency in the collaboration of healthcare professionals regarding a biopsychosocial language and treatment intensity, duration, and content. However, the implementation of guidelines and protocols has different barriers in daily practice.		
	CFIR construct	*Barrier (✗) facilitator (✓)*
**Main finding 1A: The guidelines and protocols stimulate intensive collaboration between healthcare professionals, such as consistency in the biopsychosocial language and transparency in treatment duration, intensity, and content**		
• Healthcare professionals indicate that NPRL forces collaboration in the transmural chain, which can be supported by the eHealth application.	1G/4A	✓/✗
• (Transmural) collaboration makes it more efficient to collect biopsychosocial information about the patients	1H/4B	✓
• Healthcare professionals perceive that exchanging experiences helps them to be more certain during inclusion and treatment	4B	✓
• For therapists, the collaboration with a GP and practice nurse mental health is an added value	3B/3D	✓
• Patients have often a biomedical vision towards treatment. Collaboration between healthcare professionals helps to change the mindset of the patient when healthcare professionals speak the same biopsychosocial language.	1G	✓
• Within local networks in primary care, collaboration within healthcare centers (Dutch: Gezondheidscentrums) exists more often within NPRL compared to collaboration between GPs and paramedics with separate practices	3B	✓/✗
• Due to collaboration in a local network in primary care, patients with more complex pain complaints can be treated in primary care instead of a referral to secondary or tertiary care	1C	✓
• Most advantages of NPRL are for primary care as secondary/ tertiary care practices work already with patients with CMP intensively.	1C	✗
**Main finding 1B: Healthcare professionals experience tension between a fixed protocol and the freedom to adjust the protocol into daily practice. This is influenced by their professional preferences.**		
Tension between fixed protocol and more freedom		
• Freedom is desired in the use of the treatment protocol into daily practice. Healthcare professionals underline this as an advantage	1D/1G/ 4A/5C	✓
• At the end of phase 1.1, there was a desire for more fixed protocols. Some healthcare professionals stated that it will give them more grip in this complex patient population	1G/4B	✓
• After making the treatment protocol more fixed, healthcare professionals stated that a summary of the protocol was desired. The fixed treatment protocol together with the summary were suitable in daily practice.	5C	✓
• On one hand, healthcare professionals stated that the treatment protocol must be fixed and standard when NPRL1.0 will be expanded to other regions. However, on the other hand, due to the complexity of organization of primary care in the Netherlands, it is not possible to accomplish this.	3E/1G/1F	✗
Personal preferences		
• In the treatment protocol, fewer consultations are prescribed compared to standard pain rehabilitation care in primary care. Healthcare professionals are afraid that less consultations will lead to less income	1H	✗
**Main finding 1C: Difficult to apply the guidelines about the eHealth application and assessment tools for satisfactory use in daily care.**		
eHealth application SanaCoach Pain Rehabilitation: use in daily practice		
• By using eHealth, biopsychosocial information about the patient will be collected easily which saves time during consultation and better preparation of consultation	1C	✓
• Some healthcare professionals mentioned that the eHealth application is easy to use during consultations. However, other healthcare professionals perceived the eHealth application as complex.	1G/1H/ 4B	✓/✗
• The chat function of the eHealth application does not fit with daily practice. In primary, secondary and tertiary unknown who has to keep track of the chat function and respond to patients.	3A	✗

• In healthcare several eHealth applications are used for several health conditions. Healthcare professionals have difficulties combining these different eHealth applications during consultations.	1G	✗
eHealth application SanaCoach Pain Rehabilitation: content		
• Functioning of the complete eHealth application is unknown for some healthcare professionals	4A	✗
• The eHealth application places the patient central in the treatment, which is seen as an advantage by healthcare professionals	1G	✓
• Rehabilitation physicians in secondary and tertiary care indicated that the diary function in the eHealth application had no added value as they see patients not often enough during the rehabilitation	3A	✗
eHealth application SanaCoach Pain Rehabilitation: Experiences of patients		
• Patients discussed the results in the eHealth application at the start of the treatment with the healthcare professional, but later on, this happened less	1E	✗
• The diary questionnaires are perceived as hard to fill in daily by patients. Some patients did not see the added value of it	1G/4A	✗
• Patients indicated that the graphics about daily activity are an advantage for their treatment.	1G	✓
• Overall, patients indicated the education material in the eHealth application as very useful. However, patients who were first treated in secondary or tertiary before visiting a primary healthcare professional mentioned that there is no new information in the education materials	1G	✗ / ✓
• Some patients preferred education materials on paper than integrated in an eHealth application. Not all patients have an internet connection	1G/2A	✗
• All patients agreed that the eHealth application stimulates them to adhere to the treatment	1G/4B	✓
• Some patients did not know their healthcare professional was able to view their scores in the eHealth application	1G	✗
• Patients indicated that it is difficult to get an overview of all the functions of the eHealth application	1G	✗
Assessment tool 1 primary care		
• Use of assessment tool during consultation is too time consuming	1G/1H	✗
• Healthcare professionals expect that an extra consultation to discuss the results of the assessment tool with the patient is not practical; it is an extra burden for the patient.	1G/2A	✗/✓
• Some healthcare professionals indicate that the assessment tool is not much effort for the patient, while other healthcare professionals perceive it as too much effort.	1H/4A	✓/✗
• Results of the assessment tool in primary care are not in line with their knowledge and anamnesis for some healthcare professionals. Therefore, some of them indicate that modifications are necessary while more experienced healthcare professionals indicated this is not necessary.	4A/1G	✗ / ✓
• Healthcare professionals find it important that assessment tool 1 has a good validity	1B/1G/ 4A	✓
Assessment tool 2 secondary and tertiary care		
• Healthcare professionals report that assessment tool 2 supports their knowledge and anamnesis	4B	✓
• Healthcare professionals point out that their available administrative time is not sufficient to fill in assessment tool 2	3A	✗
• After adjustments, it is easier to fill in the assessment tool, but it is still time consuming	1H/1G	✗ / ✓
Use in daily practice		
• The assessment tools and lessons learned during the education days gives the healthcare professionals more grip to coach the patient during a biopsychosocial treatment	4A	✓
• Complex to start using the assessment tool, eHealth application and contacting other involved healthcare professionals during inclusion of a new patient	1F/1G/ 5C	✗
• Because the use of the eHealth application is not an automatized process in daily care. The patient inclusion for the study is hindered as these inclusions are done via the eHealth application	3E	✗
• Assessment tools and treatment protocols make referral and treatment more consistent and objective	1C	✓
**Summary 2: Participation and implementation are hindered because of the stigmatization of CMP in society. Moreover, healthcare professionals' approaches are often more biomedical oriented instead of biopsychosocial oriented.**		

	CFIR construct	Barrier (✗) facilitator (✓)
**Main finding 2A: In the Dutch society, there is a stigma on CMP because pain is not visible.**		
• Patients as well as healthcare professionals reported an stigma on CMP in the Dutch society	2A	✗
• Healthcare professionals mentioned that diagnosing someone with CMP feels for patients as not being take serious	4A	✗
**Main finding 2B: Because of the less supported biopsychosocial vision, healthcare professionals have difficulties with (early) recognition of patients with CMP in primary care.**		
Motivation of healthcare professionals		
• Healthcare professionals' intrinsic motivation for participation is overall positive. Reasons for participation are:<br>a. Patients are not treated with evidence based care<br>b. Keep healthcare affordable<br>c. CMP is a social problem, the empowerment of these patients is important<br>d. Difficult to treat patients with CMP successfully<br>e. Increasing their personal professional network<br>f. Multidisciplinary collaboration<br>g. Earlier involved in projects with CMP<br>h. Practice owners agreed participation, healthcare professionals were obligated to participate<br>i. Trust in this scientific grounded network	1D/4A/ 4D	✓
Vision of healthcare professionals		
• Matched care is perceived as an added value for care	4A	✓
• In order to successfully recognize patients, healthcare professionals' vision must be biopsychosocial. They perceive this as difficult because they are trained with a biomedical vision so it requires more attention to screen with a biopsychosocial vision	4B	✗
• Research with questionnaires is a burden for patients	1G	✗
Knowledge of healthcare professionals		
• Participating healthcare professionals have different starting levels of knowledge regarding CMP	3D/4B	✗ / ✓
• Difficult to align the training to the different levels of knowledge regarding content and duration	1B/1G/ 5C	✗
• Some healthcare professionals need more tools to increase their certainty in treating patients with CMP.	1G/4B/ 4C	✗
• At the end of phase 1, healthcare professionals perceived the education days as confusing because the treatment protocol was not fixed, as it was developed during the meetings using input of healthcare professionals. In later phases, this became more clear	1G/4B	✗
• Education days had a clear layout	1G	✓
• Healthcare professionals perceive patients with CMP as a difficult population to manage	4B/4C	✗
• Secondary and tertiary care receives a lot of unjustified referrals from primary care, which indicates a lack of knowledge among GPs	2B/2D	✗
Recognition of patients with CMP		
• In phase 1, it was difficult to determine the level of complexity of a patient with CMP	4A	✗
• In general practice, the group of patients is diverse, which makes recognition difficult	2D	✗
• Search for the ideal patient with CMP make that healthcare professionals have other expectations and recognize only 10-20% of the CMP population	4B/3C	✗
• Healthcare professionals are afraid and insecure to make a false diagnosis of someone suspected to have CMP as they do not want to burden the patient if afterwards it might not have been necessary	4B	✗
• At the end of phase 3, healthcare professionals indicated easier recognition of patients CMP, but they still need more experience to make it a habit	5C	✗
**Main finding 2C: Healthcare professionals have difficulties with motivating patients for a biopsychosocial treatment because both their attitude is more biomedical focused**		
Vision		
• Difficult for healthcare professionals to change the biomedical vision of patients to a biopsychosocial vision	1F	✗

• In physiotherapy, patients expect a biomedical oriented therapy, with preferable massage.	3A	✗
• Some patients indicated that exercises during physiotherapy have an added value	3A	✓
• Patients who receive primary physiotherapy have not always the feeling that they can talk about their confidential CMP problem	3A	✗
Participation of patients		
• The participation of patients is lower as expected. One reason is that patients do not want to participate in a biopsychosocial treatment.	5C	✗
• If healthcare professionals are more enthusiastic, it is easier to motivate the patients for participating in the study	4B	✓
• Therapists perceive it as difficult to motivate patients, who are already visiting them for years for a biomedical treatment, for the biopsychosocial treatment. Therapist have the feeling that it lowers their credibility when they start with a 'totally different' treatment approach. Therefore, some practices choose to not include well-known patients.	3D	✗
• Not all patients are eager to participate in a study with questionnaires or eHealth	2A	✗
• The patients who are eager to participate in the biopsychosocial treatment and the research study increase the enthusiasm of the healthcare professionals	1C/1G/ 2A	✓
• Patients do not always want to switch to another healthcare professional if they have a good relationship with their current healthcare professional. Even if that treatment fits more with the level of complexity of the pain complaints.	2A/1G	
• The consumption of care is high among patients with CMP, which makes it more difficult to motivate them for a compact treatment program	2A/2B	✗
**Summary 3: The current organisation of healthcare for patients with CMP, such as the culture, structure, and financing of healthcare practices complicates the implementation between and within the practices.**		
	CFIR construct	*Barrier (✗) facilitator (✓)*
**Main finding 3A: The culture of healthcare practices, such as the ambiance and attitude, determines the success of the collaboration between healthcare professionals.**		
• A pleasant ambiance and work pressure at the work place determines someone's willingness to participate in NPRL	3A/ 3C	✓
• Current Dutch healthcare is biomedical oriented, which makes it difficult to:<br>a) Get insight into treatment protocols when a patient will be referred outside NPRL<br>b) During referral outside NPRL, the patient will get another explanation for the complaints.<br>c) Difficult to expand NPRL to other practices or regions. Not all healthcare professionals want to switch to a biopsychosocial treatment	2B/2D/ 3D	✗
• During the professional training of nearly all disciplines, only a little attention is paid to the biopsychosocial model and patients with unexplained complaints. Moreover, this information differs depending on the discipline	2D	✗
• Healthcare professionals indicate uncertainty to look further than only the biomedical part when assessment of a patient. They are afraid to miss some essential pure biomedical complaint	4B	✗
• Young and dynamic staff makes implementation easier	3D	✓
• Therapists perceive difficulties in the collaboration with GPs, which takes a lot of effort.	3B	✗
**Main finding 3B: The financing of the current organization of healthcare in the Netherlands hinders the implementation of NPRL..**		
Multidisciplinary meetings		
• It is difficult to organize multidisciplinary meetings because:<br>a) Currently, there is no existing structure for organization of multidisciplinary meetings and collaboration in Dutch primary care<br>b) In primary care, they are not financed. Healthcare professionals have to finance this by themselves, which is difficult for small practices as their buffer is small<br>c) Not every discipline is able to be physical available every time<br>d) The time pressure in primary care makes it difficult to organize enough time for meetings	1F/1H/ 2D/3A/ 3B	✗

e) For several diseases or syndromes, meetings are organized. Therefore, healthcare professionals must be available at different meetings, which takes a lot of time. Moreover, patients are often confronted with more than one disease or syndrome, which makes care more complex		
Financing of Dutch healthcare		
• Practice nurses mental health have an important role in NPRL, they support general practitioners tasks. However, in Dutch care there is not much finance available for practice nurses.	1H/2D	✗
• Healthcare professionals whish there is a bundled payments structure for primary care. As not every patient is even as complex or needs treatment in the same biopsychosocial domains. With bundled payments they assume they are better able to organize care and collaboration for patients with complex complaints	1F/2D	✗
Healthcare insurance for patients		
• In the current basic healthcare insurance, nine consultations at a therapist are financed by the insurance. Patients as well as healthcare professionals indicate that nine consultations is not enough for most patients, as the problem is more complex	1H/2A	✗
• Patients can buy additional healthcare insurance themselves. However, healthcare professionals perceive that patients with a low socioeconomic status are not able to afford this, while they need it the most	2A	✗
• Healthcare professionals are less motivated to treat patients if they already know at the start that nine consultations will not be enough	2D	✗
**Main finding 3C: The structure of the organisation of healthcare practices in primary care is complex.**		
Time pressure in primary care		
• During recruitment of healthcare practices, several practices declined participation due to lack of time	3D/3E	✗
• Participating healthcare practices are too busy to implement a new project sufficient. If busy, they work again on the automatic pilot	4B	✗
Networking in current organization of care		
• Current general practice care is unsuitable for networks on a large scale. Most GPs have their own specialization. NPRL is too complex to fit in this organization of care	1D/1E/ 2B	✗
• Multidisciplinary care in primary care is not suitable for small practices.	2B/3B	✗
• Primary care healthcare professionals are not used to referrals within their own discipline, which is necessary within NPRL	2B/2D	✗
• In current healthcare, there is no clear treatment protocol for CMP. It fits partly within different disciplines	2B/2D	✗
• Organization of primary care in the Netherlands shifts towards practice enlargements, which could be an advantage for NPRL	2D	✓
• Multidisciplinary meetings at the primary care practices stimulate the implementation of NPRL	1D/1E	✓
• If healthcare professionals within primary care are based on one address, it stimulates collaboration	3A/3D	✓
Competition		
• Competition between practices influences collaboration	1F/2B/ 2C/2D	✗
• Market forces influences referrals within primary physiotherapy, even within practices	3A	✗
• Some practice owners in physiotherapy are against selection of patients with a level of complexity which fits within primary care	3D/3E	✗
Other		
• Difficult to link ICT-systems in healthcare which barriers use of eHealth	2D	✗
• The new privacy law hinders communication between disciplines	2D	✗
**Summary 4: The iterative, bottom-up implementation strategy fits with the target audience and CMP, however, a critical mass of healthcare organisations is needed for proper implementation.**		
	CFIR construct	*Barrier (✗) facilitator (✓)*
**Main finding 4A: The active iterative, bottom-up development and participation of healthcare professionals and the project team in the implementation process of NPRL is seen as an advantage.**		
• Before the start of the project, an advisory board was started in order to discuss multidisciplinary treatment for CMP	4A	✓
• Healthcare professionals are involved in NPRL via: a) The project group b) Other participating healthcare professionals	5B	✓

c) Their practice owners<br>d) A local physiotherapist network<br>e) An advertisement		
• In research, small groups of healthcare professionals are recommended in a pilot study to implement complex interventions	1G	✓
• Healthcare professionals indicate that they were active involved in the bottom-up development of NPRL which allows adjustments which fits in daily practice	1B/1D/4D/5B/5C	✓
• Practice meetings with the project team were seen as an benefit, as they change their mindset, and are a reminder for active participation.	1G/4A/4B/5B/5C	✓
• Healthcare professionals state that the project team uses their input, has a fixed protocol, and communicates well.	1D/1G/3D/4D/5B/5C	✓
• The fact that a center of expertise is the intervention source is seen as an advantage	1A/1B/1C/4D	✓
**Main finding 4B: A critical mass of healthcare organisations is necessary for a proper executing of NPRL.**		
• In this pilot study, the amount of participating healthcare organizations was limited. This makes it difficult to collaborate and refer patients efficiently	1F/1G/3A	✗
• Practices and organizations outside NPRL are not used to multidisciplinary collaboration	3B/3D	✗
• Therapists have difficulties in collaboration for patients who visit them by direct access, because often their GP does not participate in NPRL	1G/1H/3A/3B	✗
• Within one general practice not all GPs were educated, however, sometimes a referral from within the practice itself was seen	3B	✓
• Practices within NPRL have a shared vision which makes referrals more trustful	3C	✓
**Main finding 4C: Healthcare professionals believe that NPRL is a solution to the current gap in care for patients with CMP.**		
• Healthcare professionals have the confidence that NPRL will be embedded in daily care for patients with CMP	1B/1C/1D/4A	✓
• At the end of phase 3, healthcare professionals had not a real 'network feeling' yet, but more separate practices	1D/3A	✗
• A challenge to attract healthcare professionals who are not motivated for multidisciplinary treatment or CMP	5B	✗
• Healthcare professionals have a network of colleagues, they are willing to expand NPRL within their network	3B	✓
