# Supplementary material for: Exploring the feasibility of a network of organizations for pain rehabilitation: What are the lessons learned?
Source: PLoS One. 2022 Sep 15;17(9):e0273030. doi: 10.1371/journal.pone.0273030 (PMC9477302; doi:10.1371/journal.pone.0273030)
Supplement: S1 File — (PDF) [file pone.0273030.s003.pdf]

S1 Protocol of Network Pain Rehabilitation Limburg

NPRL is described more extensively in Lamper et al. (2019), a summary is provided below:

Intervention

The main aim of Network Pain Rehabilitation Limburg was to provide integrated care for patients with CMP in order to improve their level of functioning despite pain by stimulating a biopsychosocial approach for all involved healthcare professionals. In this transmural NPRL, healthcare professionals from different disciplines and different healthcare settings were asked for participation (figure 1).

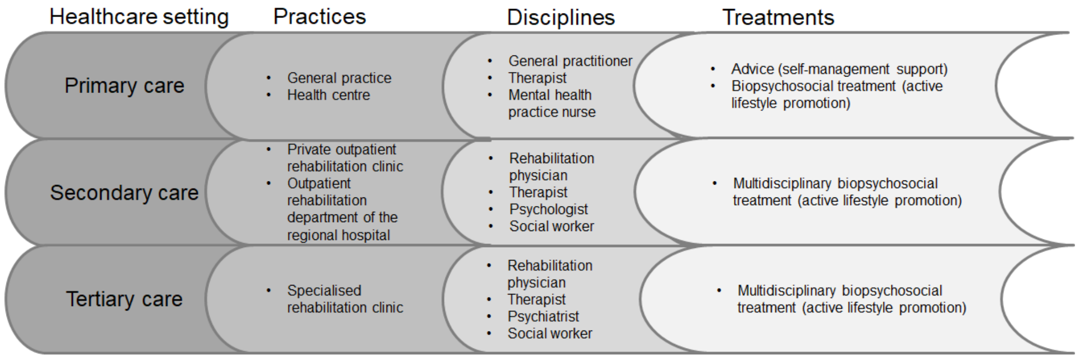

Figure 1 Construction of the health care system in Network Pain Rehabilitation Limburg. Published in Lamper et al.(2019).

Recruitment of healthcare professionals

In primary care, the recruitment started with primary care therapists or a GPs interested in pain. The interested therapists or GPs were asked to recruit a therapist or GP with whom they already have intensive collaboration. For secondary and tertiary care, main organizations in the region providing rehabilitation care for patients with CMP were asked to participate.

Setting

Each patient received the treatment needed to reach the optimal level of functioning. In order to reach this, a matched care approach was used for every individual patient. Depending on the level of disability and biopsychosocial factors involved, this either included;

(1) education only by a GP and no further treatment,
(2) monodisciplinary treatment in primary care by GP and therapy practices,
(3) multidisciplinary treatment in primary care, a collaboration between GP, primary care therapist and mental health practice nurse in assessing and treating patients with CMP who need mental support besides physical exercise,
(4) interdisciplinary treatment in secondary care in a private outpatient rehabilitation clinic,
(5) interdisciplinary treatment in tertiary care.

Collaboration was supported by facilitating communication between patients and all healthcare professionals involved in the trajectory of an individual patient by E-health. In addition, the collaboration between healthcare professionals in different practices and organizations was further supported by informative meetings, education days, an ECoach-Pain, assessment tools and treatment protocols. All healthcare professionals with different specialisms participated together in the meetings and education days. This ensures a common understanding of the biopsychosocial approach and rehabilitation treatment options.

Reference

Lamper C, Kroese M, Köke A, Ruwaard D, Verbunt J, Huijnen I. Developing the Network Pain Rehabilitation Limburg: a feasibility study protocol. BMJ Open. 2019;9(6):e025962. doi: 10.1136/bmjopen-2018-025962.
