# Supplementary material for: Exploring the feasibility of a network of organizations for pain rehabilitation: What are the lessons learned?
Source: PLoS One. 2022 Sep 15;17(9):e0273030. doi: 10.1371/journal.pone.0273030 (PMC9477302; doi:10.1371/journal.pone.0273030)

S2 Assessment Tool 1: primary care

Based and adjusted from:
Hill, J. C., et al. (2011). "Comparison of stratified primary care management for low back pain with current best practice (STarT Back): a randomised controlled trial." Lancet 378(9802): 1560-1571.
Campbell, P., et al. (2016). "Keele Aches and Pains Study protocol: validity, acceptability, and feasibility of the Keele STarT MSK tool for subgrouping musculoskeletal patients in primary care." Journal of Pain Research 9: 807-818.

1. Overall, how bothersome has your pain been in the last 2 weeks?
   O Not at all   O Slightly   O Moderately   O Very much   O Extremely

Thinking about the last 2 weeks thick you response to the following questions:

2. My pain has spread down to other parts of my body at some time in the last 2 weeks
   O Agree   O Disagree

3. I have had pain in other parts of my body than where I had pain before at some time in the last 2 weeks
   O Agree   O Disagree

4. It is not really safe for a person with a condition like mine to be physically active
   O Agree   O Disagree

5. I was bothered by my pain when executing activities at some time in the last 2 weeks
   O Agree   O Disagree

6. I have only walked sort distances because of my pain
   O Agree   O Disagree

7. Worrying thoughts have been going through my mind a lot of the time
   O Agree   O Disagree

8. I feel that my pain is terrible and it’s never going to get any better
   O Agree   O Disagree

9. In general I have not enjoyed all the things I used to enjoy
   O Agree   O Disagree

Additional information for healthcare professional:

Calculation of scores:

Question 1: *if ‘Very much’ of ‘extremely’= 1,* *Other score=0*
Question 2 t/m 9: *Agree=1,* *Diasgree=0*

Total (1 to 9) = *(score basic questions)*
Score questions 6,7,8,9 = *(psychosocial risk factors)*

‘low risk’ = Score basic questions: 0-3
The GP informs the patient with education or advice and uses the education sessions in the eHealth application.

‘medium risk’ = Score basic questions: 4-6, with 1 or 2 psychosocial risk factors
Referral of the patient to a basic exercise program with the goal to improve functioning in primary care. Executed by a physiotherapist or exercise therapist in NPRL.

‘high risk’ = Score basic questions: 6-9, with ≥ 3 psychosocial risk factors
The set A with additional questions has to be filled in by the healthcare professional in primary care.

Set A additional questions:

A1. Has the patiënt medical co-morbidity with influces daily functioning?	O Yes O No
A2. Have you been absent from work for more than 3 weeks?	O Yes O No

A3. Has the patient already undergone unsuccessful behavioral treatment for improving daily functioning in primary care?	O Yes O No

0x Yes in Set A:
Referral to behavioral treatment in primary care executed by a physiotherapist or exercise therapist in NPRL.

≥ 1 Yes in Set A:
The Set B with additional questions has to be filled in by the healthcare professional in primary care.

<u>Set B additional questions</u>:

B1. Does the patient have pain in at least 2 (independent of each other) body parts?	O Yes O No
B7. Is a patient's social environment a strong barrier to conducting behavioral treatment in primary care?<br>Eg. little or no social support and / or the presence of physical / mental stressors.	O Yes O No
B8. Has the patient sought treatment more than 3 times in the past six months (at the GP and / or physiotherapist) for the same or similar non-specific pain complaints of the musculoskeletal system?	O Yes O No
B2. Are there mood problems, not as a result of the pain complaints, that hinder the implementation of behavioral treatment in primary care?	O Yes O No
B3. Are there anxiety problems, not as a result of the pain complaints, that hinder the implementation of a behavioral treatment in primary care?	O Yes O No
B4. Are there any personality problems that hinder the implementation of behavioral treatment in primary care?	O Yes O No
B5. Are there other psychiatric problems that hinder behavioral treatment in primary care?	O Yes O No
B6. Is the psychiatric problem primarily in the foreground and is it responsible for any daily dysfunction?	O Yes O No

0x Yes in Set B:
Referral to Rehabilitation basic in NPRL (secondary care)

Question B6 = Yes:
Referral to mental healthcare

≥ 1 Yes in Set B:
Referral to medical specialist rehabilitation (tertiary care)

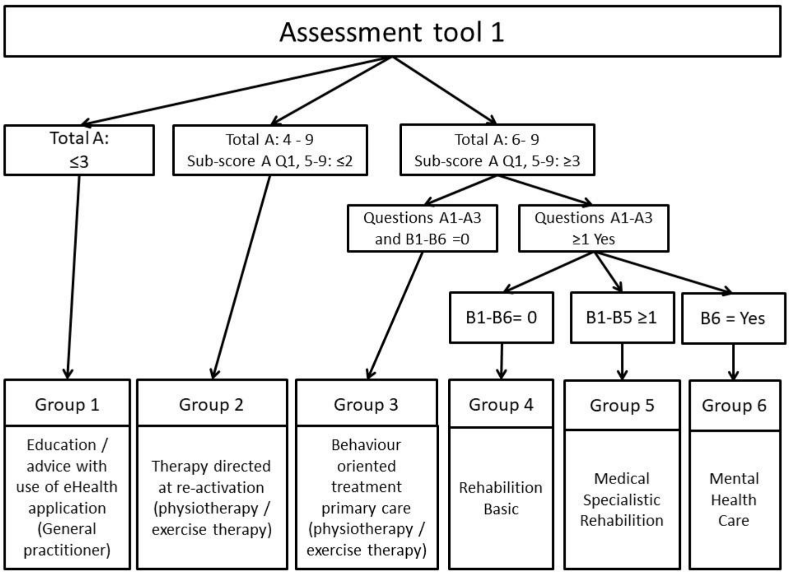

Indicatietool 1: eerste lijn

[Tekst voor de patiënt: Basis vragen]

Geef alstublieft een antwoord op elk onderdeel. Kruis bij ieder onderdeel het vakje aan dat op jou van toepassing is. Soms is het moeilijk om tussen twee vakjes te kiezen, kruis dan het vakje aan dat jouw probleem het beste beschrijft. Zou je bij het beantwoorden van onderstaande vragen willen terugdenken aan pijn **gedurende de laatste 2 weken**.

1. Over het geheel genomen, hoe hinderlijk is uw - huidige pijn - de afgelopen 2 weken geweest?

O In het geheel niet   O Een beetje   O Matig   O Erg   O Extreem

Kruis bij elk van de onderstaande vragen het hokje aan dat aangeeft of het eens of oneens bent met de stelling, terugdenkend aan de laatste 2 weken.

2. Gedurende de laatste 2 - weken heeft mijn huidige pijn zich verspreid naar ander delen van mijn lichaam

O Eens   O Oneens

3. Gedurende de laatste 2 weken heb ik soms last gehad van in andere delen van mijn lichaam dan waar ik eerst pijn had

O Eens   O Oneens

4. Het uitvoeren van lichamelijke activiteiten zou mijn herstel kunnen vertragen.

O Eens   O Oneens

5. Gedurende de laatste 2 weken werd ik bij het uitvoeren van gewone dagelijkse bezigheden belemmerd door mijn huidige pijn

O Eens   O Oneens

6. Gedurende de laatste 2 weken heb ik vanwege mijn huidige pijn alleen korte afstanden gelopen.

O Eens   O Oneens

7. Gedurende de laatste 2 weken maak ik me zorgen over mijn pijn

O Eens   O Oneens

8. Mijn huidige pijn is een groot probleem en ik geloof dat het niet meer over zal gaan

O Eens   O Oneens

9. Over het geheel genomen heb ik gedurende de laatste 2 weken niet meer genoten van dingen waarvan ik normaal wel geniet.

O Eens   O Oneens

[Tekst voor de eerstelijns verwijzer / behandelaar]

Voor verwijzing van patiënten met chronische pijn wordt in de eerste lijn, naast anamnese en lichamelijk onderzoek een screeningstool afgenomen. Op basis van de uitkomsten op een aantal anamnestische vragen worden afkappunten berekend. Deze afkappunten bepalen waar patiënten naar toe worden verwezen voor vervolgbehandeling.

Berekenen van de scores:
Het berekenen van de scores zal geïntegreerd worden in de Pijnrevalidatie Coach. Mocht je toch een vragenlijst op papier afnemen dan kun je de scores op deze manier berekenen:

Vraag 1: als 'erg' of 'extreem'= 1, anders score=0
Vraag 2 t/m 9: Eens=1, Oneens=0

Totaal (1 t/m 9) = (score basis vragen)
Score vragen 6,7,8,9 = (psychosociale risicofactoren)

'laag risico' = Score basis vragen: 0-3
De huisarts geeft patiënt voorlichting/educatie en advies, daarbij gebruikmakend van de educatie in de Pijnrevalidatie Coach.

'matig risico' = Score basis vragen: 4-6, waarvan 1 of 2 psychosociale factoren
Deze patiënt wordt doorverwezen naar een basisoefenprogramma gericht op verbeteren van dagelijks functioneren in de eerste lijn uitgevoerd door een fysiotherapeut of oefentherapeut in het netwerk.

'hoog risico' = Score basis vragen: 6-9, waarvan ≥ 3 psychosociale factoren
De set A met aanvullende vragen dient afgenomen te worden door de verwijzer/behandelaar in de eerste lijn.

Set A aanvullende vragen: [dient afgenomen te worden door de verwijzer / behandelaar in de eerste lijn]

A1. Is er sprake van medische co-morbiditeit (zoals bv pulmonale of cardiale problematiek) die het dagelijks functioneren en/of het verbeteren ervan sterk beïnvloeden?	O JA O NEE
A2. Is er sprake van arbeidsverzuim langer dan 3 weken?	O JA O NEE
A3. Heeft de patiënt al niet succesvolle gedragsgeoriënteerde behandeling voor verbeteren dagelijks functioneren in de eerste lijn doorlopen?	O JA O NEE

0x JA op Set A:
Verwijzing naar gedragsgeoriënteerde behandeling in eerste lijn door fysiotherapeut/ oefentherapeut uit netwerk.

≥ 1 JA op Set A:
De set B met aanvullende vragen dient afgenomen te worden door de verwijzer/behandelaar in de eerste lijn.

Set B aanvullende vragen: [dient afgenomen te worden door de verwijzer / behandelaar in de eerste lijn]

B1. Heeft de patiënt pijn in minstens 2 (onafhankelijk van elkaar) lichaamsdelen	O JA O NEE
B7. Is de sociale omgeving van een patiënt sterk belemmerend voor het uitvoeren van een gedragsmatige behandeling in de eerste lijn<br>Bv. geen of nauwelijks sociale steun en/of de aanwezigheid van fysieke/mentale stressoren.	O JA O NEE
B8. Heeft de patiënt het afgelopen half jaar meer dan 3x behandeling gezocht (bij de huisarts en/of fysiotherapeut) voor dezelfde of soortgelijke aspecifieke pijnklachten van het bewegingsapparaat?	O JA O NEE
B2. Is er sprake van stemmingsproblematiek, niet als gevolg van de pijnklachten, die belemmerend is voor uitvoering van een gedragsmatige behandeling in de eerste lijn?	O JA O NEE
B3.Is er sprake van angstproblematiek, niet als gevolg van de pijnklachten, die belemmerend is voor uitvoering van een gedragsmatige behandeling in de eerste lijn?	O JA O NEE
B4. Is er sprake van persoonlijkheidsproblematiek die belemmerend is voor uitvoering van een gedragsmatige behandeling in de eerste lijn?	O JA O NEE

B5. Is er sprake van andere psychiatrische problematiek die belemmerend is voor uitvoering van een gedragsmatige behandeling in de eerste lijn?	O JA O NEE
B6. Staat de psychiatrische problematiek primair op de voorgrond en is verantwoordelijk voor het eventueel aanwezige dagelijks disfunctioneren?	O JA O NEE

0x JA op Set B:
Verwijzing naar revalidatie Basis (1,5e lijn) in het Netwerk.

Vraag B6 = Ja:
Verwijzing naar de GGZ (in overleg met patiënt).

≥ 1 JA op Set B:
Verwijzing naar medisch specialistische revalidatie.

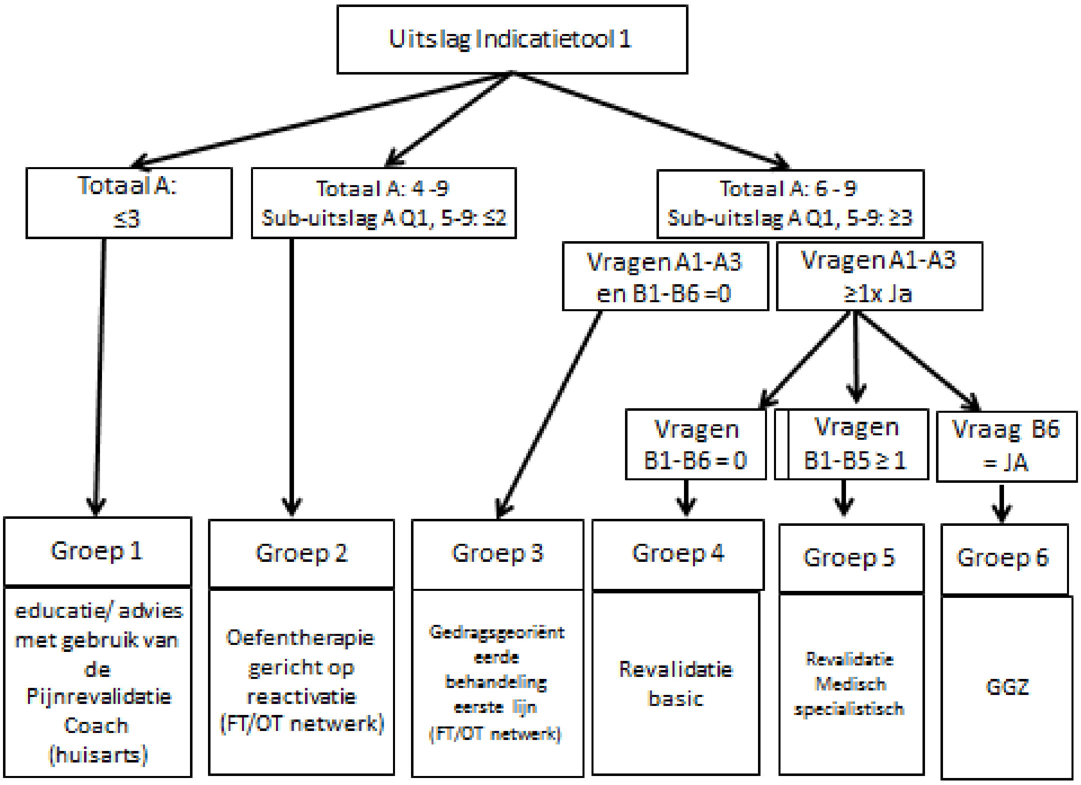

Supplement: S2 File — (PDF) [file pone.0273030.s004.pdf]
