# Supplementary material for: Exploring the feasibility of a network of organizations for pain rehabilitation: What are the lessons learned?
Source: PLoS One. 2022 Sep 15;17(9):e0273030. doi: 10.1371/journal.pone.0273030 (PMC9477302; doi:10.1371/journal.pone.0273030)
Supplement: S3 File — (PDF) [file pone.0273030.s005.pdf]

S3 Topic list focus groups and interviews

Focus groups 1&2 – phase 1 – primary care

- General opinion about NPRL
    - Adequate level of knowledge and resources
    - Added value for daily practice
    - Useability of tools
- Start meeting
    - Content
    - Alignment with daily practice
    - Way healthcare professionals are involved in NPRL
- Education meetings
    - Usefull for daily practice
    - Knowledge about CMP
- Practice meetings
    - Added value
    - Implementation of disucced topics in daily practice
- Assessment tool 1
    - Usability
    - Practical use
    - Complexity of patients
    - Integration in eHealth
- eHealth
    - Usability
    - Practical use
    - Comparison with other eHealth applications
    - Added value
- Treatment protocol
    - Usability
    - Fixed protocol in the future
    - Adaptations in protocol during education meetings
- Collaboration
    - Interdisciplinary collaboration
    - Subdividing tasks
    - Difference with usual care
- Expectations for the future
    - Implementation of NPRL in daily care
    - Implementation in your practice/organisation
    - Barriers

Focus group 3 – phase 2 – primary care

- Additional education recognition patient with CMP
    - Usability
    - Understandable
    - Added value for use in daily care
    - Need for extra education/information

- Collaboration
    - Workshops from secondary and tertiary care
        - Understandable
        - Referral to secondary and tertiary care
        - Need for extra information
    - Interdisciplinary collaboration in local network
        - Use of practice nurse mental health

- Treatment protocol
    - Summary of the protocol
    - Need for extra education/information

- eHealth
    - Extra education
    - Usability daily practice
    - Need for extra education/information
    - Preventive care

- Assessment tool
    - Usability extra rules and information
    - Integration in daily care
    - Need for extra education/information

- Network meeting with all healthcare professionals
    - General opinion
    - Points for improvement
    - Subject next edition
    - Summary of the network meeting

- Participation of patients
    - Eligible patients who did not start treatment
    - ‘Automatic process’ of inclusion and treatment

- Expectations for the future
    - What do you need?
    - Barriers
    - Adjustments

Focus group 4 – phase 2 – secondary/tertiary care

- General opinion about NPRL
    - Adequate level of knowledge and resources
    - Added value for daily practice
    - Useability of tools

- Assessment tool 2
    - Usability
    - Practical use
    - Complexity of patients
    - Patient satisfaction

- eHealth
    - Practical use
    - Comparison with other eHealth applications
    - Added value

- Collaboration
    - Primary care
    - Communication
    - Expansion with other healthcare disciplines

- Network meeting with all healthcare professionals
    - General opinion
    - Points for improvement
    - Subject next edition

- Expectations for the future
    - Better implementation in daily care
    - Barriers
    - Adjustments

Focus group 5 – phase 3 – primary, secondary and tertiary care

- Experiences in phase 2
  - Barriers
  - Facilitators

- Network meeting with all healthcare professionals
  - General opinion
  - Points for improvement
  - Subject next edition
  - Summary

- Collaboration
  - Interdisciplinary collaboration
  - Local network
  - Local networks vs secondary/tertiary care
  - eHealth and collaboration

- Network vs. usual care
  - Activities
  - Way of working

- Participation of patients
  - 'Automatic process' of inclusion and treatment
  - Barriers
  - eHealth and participation
  - Biopsychosocial model

- Assessment tool 2
  - New version
  - Usability
  - Added value

- Expectations for the future
  - Treatment of all available patients in NPRL
  - Barriers
  - Adjustments
  - Continuity NPRL inside each organization/ practice
  - Education meetings

Focus group 6 – phase 3 – patients

- General opinion about treatment and eHealth

- Treatment
    - Content of education
    - Information about treatment GP and/or therapist
    - Collaboration GP and therapist
    - Content of exercises
    - Positive points of treatment
    - Adjustment of treatment to CMP
    - Functioning and participation in daily life
    - Result
    - Recommend to family and friends

- eHealth
    - Goal
    - Interaction with therapist
    - Content of the application
    - Giving control over treatment
    - Recommend to family and friends

- Assessment tool / questionnaire
    - General opinion
    - Need of help of relatives
    - Which moment in the treatment

- Referral
    - Why and to which discipline
    - Opinion referral
    - Waiting times
    - Information given
    - Alignment with CMP

- Social environment
    - Involvement of family or friends in treatment and eHealth
    - Opinion about treatment and eHealth

Interview 1 – phase 1 – RP – tertiary care

- Current organisation of care
    - Barriers
    - Facilitators
    - Management

- Collaboration with primary care
    - Referrals

- Collaboration with other departments in same organization
    - Use of questionnaires

- Content of NPRL
    - Expectations for future implementation
    - NPRL solution for barriers of care as usual

Interview 2 – phase 1 - RP – tertiary care

- Assessment tool 2
    - General opinion
    - Comparison with care as usual
    - Opinion of the team
    - Practical barriers

- Current organisation of care
    - Barriers
    - Facilitators
    - Referral patterns

- Content of NPRL
    - Opinion about participation of commercial organization of pain rehabilitation
- Collaboration with primary care
    - Referrals

- Collaboration with other departments in same organization

Interview 3 – phase 1 – psychologist – secondary care

- Current organisation of care
    - Barriers
    - Facilitators
    - Referral patterns
    - Management

- Content of NPRL
    - Expectations for future implementation
    - NPRL solution for barriers of care as usual

- Assessment tool 2
    - Barriers
    - Adjustments in daily practice

- eHealth
    - Expectations
    - Adjustments in daily practice

- Collaboration
    - Primary care
    - Other organizations
    - Influence of NPRL

Interview 4 – phase 2 – RP – secondary care

- Current organisation of care
    - Barriers
    - Facilitators
    - Referral patterns
    - Management

- Content of NPRL
    - Expectations for future implementation
    - NPRL solution for barriers of care as usual
    - Expected added value

- Assessment tool 2
    - Barriers
    - Adjustments in daily practice

- eHealth
    - Expectations
    - Adjustments in daily practice

- Collaboration
    - Primary care
    - Other organizations
    - Influence of NPRL

Interview 5 – phase 2 - practice nurse mental health – primary care

- Network meeting with all healthcare professionals
    - General opinion
    - Points for improvement

- Collaboration
    - Facilitators
    - Local network
    - Comparison with usual care

- Treatment protocol
    - Specific for practice nurse mental health
    - Content

- eHealth
    - Experiences
    - Feedback system
    - Collaboration

- Participation of patients
    - Financial situation
    - Satisfaction

- Transferability of NPRL
    - Healthcare disciplines

    - Local network

Interview 6 – phase 2 – RP – secondary care

- Current organisation of care
    - Barriers
    - Facilitators
    - Referral patterns
    - Management

- Content of NPRL
    - Expectations for future implementation
    - NPRL solution for barriers of care as usual
    - Expected added value

- Assessment tool 2
    - Barriers
    - Adjustments in daily practice

- eHealth
    - Expectations
    - Adjustments in daily practice

- Collaboration
    - Primary care
    - Other organizations
    - Influence of NPRL
