# Supplementary material for: Exploring the feasibility of a network of organizations for pain rehabilitation: What are the lessons learned?
Source: PLoS One. 2022 Sep 15;17(9):e0273030. doi: 10.1371/journal.pone.0273030 (PMC9477302; doi:10.1371/journal.pone.0273030)
Supplement: Attachment — Submitted filename: Rebuttal_ExpFeasNetwOrgPainRehab_May2022.pdf [file pone.0273030.s007.pdf]

Jianhong Zhou
Staff Editor
PLOS ONE

Maastricht, the Netherlands, May 18, 2022

Manuscript title: Exploring the feasibility of a network of organizations for pain rehabilitation: what are the lessons learned?
Manuscript ID: PONE-D-20-32156

Dear Jianhong Zhou,

Thank you very much for considering our paper "Exploring the feasibility of a network of organizations for pain rehabilitation: what are the lessons learned?" for PLOS ONE and for having it reviewed a third reviewer in the field of chronic pain. We have carefully considered the journal requirements and reviewers' comment, and made the revision to the paper as suggested. We appreciate the helpful feedback that was provided and believe that the advice to add an extra supporting file was relevant and useful to improve the understandability of our manuscript. The changes in the text are visible by track changes. Please find our reaction to the reviewer and summary of the adaptations below. Our reactions are indicated with symbol '>'.

As asked by the third reviewer, we have added an extra Supporting file with a summary of our published protocol article for clarification.

We hope we have met all requirements for publication of the manuscript and we hope to receive a positive response to our re-submission soon..

On behalf of all authors,

Yours sincerely,

Cynthia Lamper, MSc

Maastricht University
Faculty of Health, Medicine and Life Sciences
Department of Rehabilitation Medicine
P.O. Box 616, 6200 MD Maastricht
The Netherlands
E-mail: cynthia.lamper@maastrichtuniversity.nl

<u>Journal requirements and reviewer(s)' Comments to Author:</u>

Comment 1: Please review your reference list to ensure that it is complete and correct. If you have cited papers that have been retracted, please include the rationale for doing so in the manuscript text, or remove these references and replace them with relevant current references. Any changes to the reference list should be mentioned in the rebuttal letter that accompanies your revised manuscript. If you need to cite a retracted article, indicate the article's retracted status in the References list and also include a citation and full reference for the retraction notice.

> We have checked all references and they are all still existing.

<u>Reviewer 3:</u>

Comment 1: The study was extensively revised. However, to make it more understandable in my opinion it could be useful to add as supporting information what is written in the published protocol: in particular, the explanation of the primary, secondary and tertiary care with the short related protocol.

> We thank reviewer 3 for this comment. We agree that an explanation of the healthcare lines and content of NPRL is of added value for the understandability of our study. Therefore, we have added a summary of our published protocol in Supplementary file 1.We adjusted L124 with a referral to the Supporting file.

L124. NPRL is described more extensively in Lamper et al. (2019) (a summery can be found in Supporting file 1): it was developed by a project team consisting of the authors CL, IH, AK, JV and an advisory board consisting of interested HCPs
