# Supplementary material for: Exploring the feasibility of a network of organizations for pain rehabilitation: What are the lessons learned?
Source: PLoS One. 2022 Sep 15;17(9):e0273030. doi: 10.1371/journal.pone.0273030 (PMC9477302; doi:10.1371/journal.pone.0273030)
Supplement: Attachment — Submitted filename: Rebuttal_ExpFeasNetwOrgPainRehab_July2022.pdf [file pone.0273030.s008.pdf]

Jianhong Zhou
Staff Editor
PLOS ONE

Maastricht, the Netherlands, July 21, 2022

Manuscript title: Exploring the feasibility of a network of organizations for pain rehabilitation: what are the lessons learned?
Manuscript ID: PONE-D-20-32156

Dear Jianhong Zhou,

Thank you very much for considering our paper "Exploring the feasibility of a network of organizations for pain rehabilitation: what are the lessons learned?" for PLOS ONE and for having it reviewed a third reviewer in the field of chronic pain. Please find our reaction to the reviewer and summary of the adaptations below. Our reactions are indicated with symbol '>'.

We have added the study registration data on the title page of the manuscript. The study is registered at the Netherlands Trial Register at 18/08/2017 (Registration number: NTR6654 or https://trialsearch.who.int/Trial2.aspx?TrialID=NTR6654).

We hope we have met all requirements for publication of the manuscript and we hope to receive a positive response to our re-submission soon.

On behalf of all authors,

Yours sincerely,

Cynthia Lamper, MSc

Maastricht University
Faculty of Health, Medicine and Life Sciences
Department of Rehabilitation Medicine
P.O. Box 616, 6200 MD Maastricht
The Netherlands
E-mail: cynthia.lamper@maastrichtuniversity.nl

__**Journal requirements and reviewer(s)' Comments to Author:**__

**Comment 1: Specifically, the in-house editorial staff feels that your study should be registered as a clinical trial. In particular, the participants were subject to changes in treatment approach, and health related outcomes were collected as part of the study. There is no need to provide clinical trial related documents (e.g. trial protocol or the CONSORT flow diagram) and to report the work as a clinical trial (i.e. no need to change article type into "clinical trial"), but we do request having your study registered before we proceed.**

> We have added the study registration data on the title page of the manuscript. The study is registered at the Netherlands Trial Register at 18/08/2017 (Registration number: NTR6654 or https://trialsearch.who.int/Trial2.aspx?TrialID=NTR6654).

__**Reviewer 3:**__

**Comment 1: The revised form of this study has been extensively modified. In this way in my opinion the study can be published**

> We thank reviewer 3 for this comment.
